# Supplementary material for: Single-cell Atlas of common variable immunodeficiency shows germinal center-associated epigenetic dysregulation in B-cell responses
Source: Nat Commun. 2022 Apr 1;13:1779. doi: 10.1038/s41467-022-29450-x (PMC8975885; doi:10.1038/s41467-022-29450-x)
Supplement: Supplementary file 2 — Description of Additional Supplementary Files [file 41467_2022_29450_MOESM2_ESM.pdf]

DESCRIPTION OF ADDITIONAL SUPPLEMENTARY FILES

Supplementary Data 1. Clinical information and B cell phenotype of the CVID patients and healthy donors included in the study, as well as vaccination response of CVID-discordant twins.

Supplementary Data 2. Stats Mapping Hiseq DNA methylation.

Supplementary Data 3. Genetic analysis of the CVID-discordant MZ twins.

Supplementary Data 4. Differentially methylated regions identified in the transition from naïve BCs to US-mem and S-mem Bcs.

Supplementary Data 5. CVID.no-demeth DMRs

Supplementary Data 6. Gene expression analysis (CVID twin vs Control twin) of the different B cell compartments (naïve, US-mem and S-mem B cells) using SS2 data.

Supplementary Data 7. Differentially expressed genes (adj.p-value < 0.05) in the CVID twin compared with the control twin in the different cell compartments.

Supplementary Data 8. CellPhoneDB analysis in the CVID-discortant twins.

Supplementary Data 9. Amplicon-sequencing. Watson or Crick strands (WC.strand), chromosome (Chr), number of methylated reads (reads.ME), number of unmethylated reads (reads.UM), percentage of methylation (percent.ME).

Supplementary Data 10. Differentially expressed genes (adj. P-value < 0.05) in CVID patients compared with control donors in the different cell compartments.

Supplementary Data 11. Differentially expressed genes (adj. P-value < 0.05) in CVID Ib patients compared with control donors in the different cell compartments.

Supplementary Data 12. List of the different antibodies used for CITE-seq analysis.

Supplementary Data 13. Differentially expressed proteins (adj.P.Val < 0.05) in CVID patients compared with control donors in the different cell compartments.

Supplementary Data 14. Differentially expressed proteins (adj.P.Val < 0.05) in CVID Ib patients compared with control donors in the different cell compartments.

Supplementary Data 15. CellPhoneDB analysis in the cohort of CVID patients and healthy donors.
