# Supplementary material for: Single-cell Atlas of common variable immunodeficiency shows germinal center-associated epigenetic dysregulation in B-cell responses
Source: Nat Commun. 2022 Apr 1;13:1779. doi: 10.1038/s41467-022-29450-x (PMC8975885; doi:10.1038/s41467-022-29450-x)
Supplement: Supplementary file 18 — Reporting Summary [file 41467_2022_29450_MOESM18_ESM.pdf]

nature research

Corresponding author(s): Javier Rodríguez-Ubreva, Roser Vento-Tormo, Esteban Ballestar

Last updated by author(s): Dec 20, 2021

Reporting Summary

Nature Research wishes to improve the reproducibility of the work that we publish. This form provides structure for consistency and transparency in reporting. For further information on Nature Research policies, see our Editorial Policies and the Editorial Policy Checklist.

Statistics

For all statistical analyses, confirm that the following items are present in the figure legend, table legend, main text, or Methods section.

n/a	Confirmed	
☐	☒	The exact sample size (*n*) for each experimental group/condition, given as a discrete number and unit of measurement
☐	☒	A statement on whether measurements were taken from distinct samples or whether the same sample was measured repeatedly
☐	☒	The statistical test(s) used AND whether they are one- or two-sided<br>*Only common tests should be described solely by name; describe more complex techniques in the Methods section.*
☒	☐	A description of all covariates tested
☐	☒	A description of any assumptions or corrections, such as tests of normality and adjustment for multiple comparisons
☐	☒	A full description of the statistical parameters including central tendency (e.g. means) or other basic estimates (e.g. regression coefficient) AND variation (e.g. standard deviation) or associated estimates of uncertainty (e.g. confidence intervals)
☐	☒	For null hypothesis testing, the test statistic (e.g. *F*, *t*, *r*) with confidence intervals, effect sizes, degrees of freedom and *P* value noted<br>*Give P values as exact values whenever suitable.*
☒	☐	For Bayesian analysis, information on the choice of priors and Markov chain Monte Carlo settings
☒	☐	For hierarchical and complex designs, identification of the appropriate level for tests and full reporting of outcomes
☐	☒	Estimates of effect sizes (e.g. Cohen's *d*, Pearson's *r*), indicating how they were calculated

Our web collection on statistics for biologists contains articles on many of the points above.

Software and code

Policy information about availability of computer code

Data collection	Software/packages used include: R (v4.0.3), Rstudio (v1.2.5033), Python 3, Scanpy, HISAT2, 10X Genomics' Cell Ranger, HTSeq-count, cisTopic, PLINK (v1.9), Adobe Illustrator, FlowJo. Detailed parameters of each of the methods are mentioned in relevant sections in Methods.
Data analysis	All code used to perform the analysis can be found at: https://github.com/ventolab/CVID/<br>Software/packages used include: R, Rstudio, Python 3, Scanpy, HISAT2, 10X Genomics' Cell Ranger, HTSeq-count, cisTopic, PLINK, Adobe Illustrator, FlowJo. Detailed parameters of each of the methods are mentioned in relevant sections in Methods.

For manuscripts utilizing custom algorithms or software that are central to the research but not yet described in published literature, software must be made available to editors and reviewers. We strongly encourage code deposition in a community repository (e.g. GitHub). See the Nature Research guidelines for submitting code & software for further information.

Data

Policy information about availability of data

All manuscripts must include a data availability statement. This statement should provide the following information, where applicable:

- Accession codes, unique identifiers, or web links for publicly available datasets
- A list of figures that have associated raw data
- A description of any restrictions on data availability

All the data generated in this study as well as interactive visualizations of single-cell transcriptomic datasets from this study can be accessed via https://single-cell-atlas-cvid.cellgeni.sanger.ac.uk/.
WGS results are not publicly available due to ethical regulations (patients anonymization), but are available from the corresponding authors upon request.
RNA-seq and ChIP-seq publicly-available datasets for naïve-, GC, S-mem B cells, as well as for PCs from healthy donors were obtained from the Blueprint Consortium (http://dcc.blueprint-epigenome.eu/#/datasets), with the following accession numbers: Naïve BCs RNAseq (EGAD00001002315), GC BCs RNAseq

(EGAD00001002452), S-mem BCs RNAseq (EGAD00001002476), Plasma cells RNAseq (EGAD00001002323), Naïve BCs ChIPseq (EGAD00001002466), GC BCs ChIPseq (EGAD00001002442), S-mem BCs ChIPseq (EGAD00001002430), Plasma cells ChIPseq (EGAD00001002281).
We used additional ChIP-seq publicly-available datasets for several transcription factors with the following accession numbers: CTCF from human B cells (GSM1003474), as well as JUNB (GSE96455), BATF (GSM803538), CTCF (ENCSR184YZV) and PAX8 (GSE127505) from the GM12878 human lymphoblastoid cell line.
Genome Reference Consortium Human Build 38 patch release 12 (GRCh38p12).

All the code used in the analyses can be found at https://github.com/ventolab/CVID.

Field-specific reporting

Please select the one below that is the best fit for your research. If you are not sure, read the appropriate sections before making your selection.

☒ Life sciences ☐ Behavioural & social sciences ☐ Ecological, evolutionary & environmental sciences

For a reference copy of the document with all sections, see nature.com/documents/nr-reporting-summary-flat.pdf

Life sciences study design

All studies must disclose on these points even when the disclosure is negative.

Sample size	The sample size was not predetermined and all samples that were available were processed.
Data exclusions	No exclusion was applied to the uploaded raw data in ArrayExpress. For the final count matrix, we excluded cells based on pre-established criteria for single-cells: we excluded low quality samples and contaminating cells (i.e. - cells with low number of detected genes and high mitochondria content) - exclusion criteria for each case are comprehensively detailed in the relevant Methods section.
Replication	Some of the most relevant findings in the CVID-discordant twins comparison were validated in an additional cohort of CVID patients and healthy donors. Due to sample availability, no replication using the same samples was performed.
Randomization	The balance in sex and age covariates were tested in the two groups of comparison and non significant differences were found.
Blinding	Blinding is not relevant for this study as the aim is describing differences between healthy donors and CVID patients. Blinding does not affect the interpretation of the data given that we only focus on statistical significant differences.

Reporting for specific materials, systems and methods

We require information from authors about some types of materials, experimental systems and methods used in many studies. Here, indicate whether each material, system or method listed is relevant to your study. If you are not sure if a list item applies to your research, read the appropriate section before selecting a response.

Materials & experimental systems

n/a	Involved in the study	
☐	☒	Antibodies
☒	☐	Eukaryotic cell lines
☒	☐	Palaeontology and archaeology
☒	☐	Animals and other organisms
☐	☒	Human research participants
☒	☐	Clinical data
☒	☐	Dual use research of concern

Methods

n/a	Involved in the study	
☒	☐	ChIP-seq
☐	☒	Flow cytometry
☒	☐	MRI-based neuroimaging

Antibodies

Antibodies used	CD19-FITC (BD Biosciences, clone: 4G7. Cat. No. 15856028). Flow cytometry 2uL:1 million cells. Supplementary Figure 2a,b<br>CD27-APC (Miltenyi Biotec, clone: M-T271. Cat. No. 130-113-630). Flow cytometry 2uL:1 million cells. Supplementary Figure 2a,b<br>IgD-PE (Southern Biotech, Ref: 2032-09, clone: N/A).Flow cytometry 2uL:100 million cells. Supplementary Figure 2a,b<br>CD40L-PE (Becton Dickinson, Ref: 340477, clone: 89-76).Flow cytometry 2uL:1 million cells. Supplementary Figure 6d<br>CD4-APC (Miltenyi Biotec, Ref: 130-113-222, clone:REA623). Flow cytometry 2uL:1 million cells. Supplementary Figure 6d<br>TotalSeq™-C Human Uniersal Cocktail, V1.0, Biolegend (List of antibodies and their clones in Supplementary Data 12)
Validation	CD19-FITC Flow cytometry (Routinely Tested) . Flow cytometric analysis of CD19 expression on human peripheral blood lymphocytes (website).<br>CD27-APC Flow cytometry (Routinely Tested) . Flow cytometric analysis of CD27 expression on human peripheral blood lymphocytes (website).<br>IgD-PE Flow cytometry (Routinely Tested) . Flow cytometric analysis of IgD expression on human peripheral blood lymphocytes (website).

CD40L-PE Flow cytometry (Routinely Tested) . Flow cytometric analysis of CD40L on phorbol 12-myristate 13-acetate (PMA) and ionomycin-activated PBMCs (website).
CD4-APC Flow cytometry (Routinely Tested) . Flow cytometric analysis of CD4 expression on human peripheral blood lymphocytes (website).
We validated the TotalSeq™-C Human Uniersal Cocktail, V1.0, Biolegend, to use 1 vial per 1 million cells.

Human research participants

Policy information about studies involving human research participants

Population characteristics	The study was conducted using peripheral blood samples isolated from a pair of male, monozygotic twins discordant for CVID, as well as an additional cohort of CVID patients and healthy donors. See Supplementary Table 1 for details about specific clinical and immune characteristics of the donors.
Recruitment	CVID patients were diagnosed according to European Society for Immunodeficiencies (ESID) criteria. They were collected during regular medical appoitments at the University Hospital Dr Negrín, Gran Canaria, Spain and at the Hospital La Paz, Madrid, Spain. Healthy donors were collected matching in age and sex with CVID patients, so any bias that could affect the results is present.
Ethics oversight	All donors received oral and written information about the possibility that their blood would be used for research purposes, and any questions that arose were then answered. Before giving their first blood sample the donors signed a consent form approved by the Ethics Committee at their corresponding hospital (Hospital La Paz PI-2833), which adhered to the principles set out in the WMA Declaration of Helsinki. The protocol used to isolate B cells from these donors was approved by the Ethics Committee of the Bellvitge University Hospital (CEIC) on 9 March 2017 (PR053/17).

Note that full information on the approval of the study protocol must also be provided in the manuscript.

Flow Cytometry

Plots

Confirm that:

☒ The axis labels state the marker and fluorochrome used (e.g. CD4-FITC).

☒ The axis scales are clearly visible. Include numbers along axes only for bottom left plot of group (a 'group' is an analysis of identical markers).

☒ All plots are contour plots with outliers or pseudocolor plots.

☒ A numerical value for number of cells or percentage (with statistics) is provided.

Methodology

Sample preparation	PBMCs were obtained from peripheral blood by Ficoll gradient using LymphoprepTM (Stemcell Technologies. Cat. No. # 07801). For the isolation of CD19+ CD27neg IgD+ naïve, CD19+ CD27+ IgD+ unswitched memory (US-mem) and CD19+ CD27+ IgDneg switched memory (S-mem) B cells, PBMCs were stained with anti-CD19-FITC (BD Biosciences, clone: 4G7. Cat. No. 15856028), anti-CD27-APC (Miltenyi Biotec, clone: M-T271. Cat. No. 130-113-630) and anti-IgD-PE (Southern Biotech, Birmingham, AL, USA, clone: NA, Ref: 2032-09) in MACS buffer (PBS + 2% FBS + 2 mM EDTA).
Instrument	Flow cytometry was performed in a Gallios Flow Cytometer (Beckman Coulter, CA, USA). Cells were sorted in a Beckman Coulter MoFlo Astrios EQ Cell Sorter instrument.
Software	Flowjo software v9
Cell population abundance	The purity of naïve B cells within post-sort fraction (> 95%) was determined analyzing the sorted cells in a cytometer instrument just after their sorting. Due to the scarcity of memory B cells (less than 1% of total cells), testing their purity was not feasible.
Gating strategy	B cell subsets (naïve, Un-switched memory and Switched memory B cells) were sorted as follow: Singlets (SSC-Width/SSC-Area gate and Hoechst 33342-Height/Hoechst 33342-Area gate), viable cells (7AAD negative gate), lymphocytes (SSC-Area and FCSArea gate), B cells (CD19-FITC+ gate) and resting cells (gate on the 2n peak using Hoechst 33342-Height). Finally naïve (CD27- APCneg IgD-PE+), Un-switched memory (CD27-APC+ IgD-PE+) and Switched memory (CD27-APC+ IgD-PEneg) B cells were gated. Supplementary Figure 2.

☒ Tick this box to confirm that a figure exemplifying the gating strategy is provided in the Supplementary Information.
